# The accuracy and robustness of plasma biomarker models for amyloid PET positivity

**DOI:** 10.1186/s13195-021-00942-0

**Published:** 2022-02-07

**Authors:** Andréa L. Benedet, Wagner S. Brum, Oskar Hansson, Alzheimer’s Disease Neuroimaging Initiative, Thomas K. Karikari, Eduardo R. Zimmer, Henrik Zetterberg, Kaj Blennow, Nicholas J. Ashton

**Affiliations:** 1 Department of Psychiatry and Neurochemistry, Institute of Neuroscience and Physiology, The Sahlgrenska Academy, University of Gothenburg, Gothenburg, Sweden; 2 Translational Neuroimaging Laboratory, McGill Centre for Studies in Aging, McGill University, Montreal, Quebec, Canada; 3 Graduate Program in Biological Sciences: Biochemistry, Universidade Federal do Rio Grande do Sul (UFRGS), Porto Alegre, Brazil; 4 Clinical Memory Research Unit, Department of Clinical Sciences, Malmö, Lund University, Lund, Sweden; 5 Memory Clinic, Skåne University Hospital, Malmö, Sweden; 6 Department of Psychiatry, University of Pittsburgh, Pittsburgh, PA USA; 7 Department of Pharmacology, UFRGS, Porto Alegre, Brazil; 8 Graduate Program in Biological Sciences: Pharmacology and Therapeutics, UFRGS, Porto Alegre, Brazil; 9 Clinical Neurochemistry Laboratory, Sahlgrenska University Hospital, Mölndal, Sweden; 10 Department of Neurodegenerative Disease, UCL Institute of Neurology, London, UK; 11 UK Dementia Research Institute at UCL, London, UK; 12 Hong Kong Center for Neurodegenerative Diseases, Hong Kong, China; 13 Wallenberg Centre for Molecular and Translational Medicine, Department of Psychiatry and Neurochemistry, Institute of Neuroscience and Physiology, the Sahlgrenska Academy at the University of Gothenburg, Gothenburg, Sweden; 14 King’s College London, Institute of Psychiatry, Psychology & Neuroscience, Maurice Wohl Clinical Neuroscience Institute, London, UK; 15 NIHR Biomedical Research Centre for Mental Health & Biomedical Research Unit for Dementia at South London & Maudsley NHS Foundation, London, UK

**Keywords:** Amyloid, Plasma biomarker, Mass spectrometry, Immunoassay, Alzheimer’s disease, ADNI, p-tau181, GFAP, NfL

## Abstract

**Background:**

Plasma biomarkers for Alzheimer’s disease (AD) have broad potential as screening tools in primary care and disease-modifying trials. Detecting elevated amyloid-β (Aβ) pathology to support trial recruitment or initiating Aβ-targeting treatments would be of critical value. In this study, we aimed to examine the robustness of plasma biomarkers to detect elevated Aβ pathology at different stages of the AD continuum. Beyond determining the best biomarker—or biomarker combination—for detecting this outcome, we also simulated increases in inter-assay coefficient of variability (CV) to account for external factors not considered by intra-assay variability. With this, we aimed to determine whether plasma biomarkers would maintain their accuracy if applied in a setting which anticipates higher variability (i.e., clinical routine).

**Methods:**

We included 118 participants (cognitively unimpaired [CU, *n* = 50], cognitively impaired [CI, *n* = 68]) from the ADNI study with a full plasma biomarker profile (Aβ42/40, GFAP, p-tau181, NfL) and matched amyloid imaging. Initially, we investigated how simulated CV variations impacted single-biomarker discriminative performance of amyloid status. Then, we evaluated the predictive performance of models containing different biomarker combinations, based both on original and simulated measurements. Plasma Aβ42/40 was represented by both immunoprecipitation mass spectrometry (IP-MS) and single molecule array (Simoa) methods in separate analyses. Model selection was based on a decision tree which incorporated Akaike information criterion value, likelihood ratio tests between the best-fitting models and, finally, and Schwartz’s Bayesian information criterion.

**Results:**

Increasing variation greatly impacted the performance of plasma Aβ42/40 in discriminating Aβ status. In contrast, the performance of plasma GFAP and p-tau181 remained stable with variations >20%. When biomarker models were compared, the models “AG” (Aβ42/40 + GFAP; AUC = 86.5), “A” (Aβ42/40; AUC = 82.3), and “AGP” (Aβ42/40 + GFAP + p-tau181; AUC = 93.5) were superior in determining Aβ burden in all participants, within-CU, and within-CI groups, respectively. In the robustness analyses, when repeating model selection based on simulated measurements, models including IP-MS Aβ42/40 were also most often selected. Simoa Aβ42/40 did not contribute to any selected model when used as an immunoanalytical alternative to IP-MS Aβ42/40.

**Conclusions:**

Plasma Aβ42/40, as quantified by IP-MS, shows high performance in determining Aβ positivity at all stages of the AD continuum, with GFAP and p-tau181 further contributing at CI stage. However, between-assay variations greatly impacted the performance of Aβ42/40 but not that of GFAP and p-tau181. Therefore, when dealing with between-assay CVs that exceed 5%, plasma GFAP and p-tau181 should be considered for a more robust determination of Aβ burden in CU and CI participants, respectively.

**Supplementary Information:**

The online version contains supplementary material available at 10.1186/s13195-021-00942-0.

## Introduction

Therapies targeting amyloid beta (Aβ), a defining feature in the pathophysiology of Alzheimer’s disease (AD) [1], have recently been developed and proven to reduce Aβ plaque load in the brain [2–5]. However, the cognitive benefit to symptomatic patients is either very mild or, in most cases, inconclusive. The reasons for these findings are unclear, but it is hypothesized that anti-Aβ trials target a population too advanced in the disease course or that the trial duration does not have the length to observe a conclusive cognitive benefit. Nonetheless, therapeutic trials that target any phase of the AD continuum require confirmatory evidence of Aβ burden—which is of principal importance in trials that will target preclinical AD. Cerebrospinal fluid (CSF) Aβ42/40 and Aβ positron emission tomography (PET) imaging are highly representative of Aβ burden, and the latter is likely a fundamental obligation to prove target engagement throughout an intervention trial. Still, neither CSF nor PET biomarkers have the capacity to serve as a population screening tool for eligibility to anti-Aβ trials.

A blood biomarker would act as a widely accessible and simplified triage of large and diverse populations to indicate appropriate individuals for therapeutic trial recruitment—irrespective of disease stage. Furthermore, in a clinical setting, an indication that mild cognitive symptoms are accompanied by Aβ pathology is of importance for the specialist delivering a diagnosis and symptomatic treatment and, soon, determining which disease-modifying treatment would be more suitable. The development of plasma biomarkers has been driven by targeting candidates proven to be successful in CSF. Novel mass spectrometry and ultra-sensitive immunoassay methods have recently allowed for the measurement of the Aβ42/Aβ40 ratio and concentrations of phosphorylated tau (p-tau), glial fibrillary acidic protein (GFAP), and neurofilament light (NfL) in blood.

In this context, plasma Aβ42/40 has been shown to be associated with CSF and PET measures of Aβ and to be capable of identifying Aβ-positive individuals with high accuracy [6, 7]. However, this is suggested to be assay-dependent given the emerging data highlighting the superior accuracy of immunoprecipitation mass spectrometry (IP-MS) compared with ultrasensitive immunoassays for the detection of cerebral Aβ [8]. In contrast, immunoassays for the detection of p-tau181 (as well as other epitopes; p-tau217 [9] and p-tau231 [10]) in plasma have been shown to be most valuable in identifying AD in heterogeneous dementia population [11–14] and in predicting cognitive decline [11, 15, 16], besides also being highly correlated with cerebral Aβ burden. GFAP, a biomarker of astrocyte reactivity, increases in preclinical AD and is a promising plasma biomarker for this stage of the disease [17–19]. While CSF GFAP is seemingly associated with Aβ pathology only in symptomatic individuals, plasma GFAP continues to rise during disease evolution in parallel with clinical syndrome severity and Aβ accumulation [17, 19]. These recent findings suggest that plasma GFAP is more closely related to abnormal Aβ accumulation due to AD, whereas CSF GFAP may also incorporate changes independent of Aβ pathology. Increases in plasma NfL are a widely reported finding in AD [20, 21] and are also observed in pre-symptomatic familial AD [22]. Contrasting to Aβ and p-tau, NfL is not specific to AD pathology and is increased in many other neurodegenerative disorders [23] and acute neurological conditions [24]. Hence, plasma biomarkers for AD are either directly (Aβ42/40) or indirectly (e.g., tau phosphorylation, astrocyte reactivity and neurodegeneration) associated with presence of Aβ pathology and could be used to indicate elevated Aβ burden for therapeutic trials. They could be used as standalone tests or in a combinational biomarker panel, but different configurations and accuracies will likely depend on disease stage; Aβ42/40 and GFAP are likely to be more associated with preclinical Aβ, whereas p-tau181 and NfL may be later markers with increases more apparent in the transition between preclinical and prodromal AD.

In this brief report, we studied the available plasma biomarker results from the Alzheimer Disease Neuroimaging Initiative (ADNI), Aβ42/40, p-tau181, GFAP, and NfL, to suggest which biomarker(s) models would be best suited as a population prescreen for Aβ burden in a clinically heterogeneous population (i.e., all participants independent of disease stage), composed by cognitively unimpaired (CU) participants and cognitively impaired (CI) patients. Further, we sought to determine the robustness of single or multi-biomarker models to identify Aβ burden by assessing whether simulated changes in biomarker concentration (0–20%) values would significantly impact on the predictive power or model selection.

## Methods

### Study participants

We used data from the multicenter ADNI study, designed to develop and validate neuroimaging and biochemical biomarkers for the early detection, monitoring, and treatment of AD, and its inclusion criteria have been further described elsewhere [25]. All enrolled participants or authorized representatives provided informed consent, approved by ADNI center’s respective Institutional Review Boards. For this study, we included participants based on the availability of Aβ PET and full plasma biomarker profiles [Aβ42/40 (Washington University—IP-MS), p-tau181 (University of Gothenburg), GFAP (Simoa Neuro 4-plex E), and NfL (Simoa Neuro 4-plex E)]. Duplicate measurements of plasma biomarkers were excluded (*n* = 9), leading to a final sample of *n* = 118 participants. Following ADNI’s diagnostic criteria, subjects clinically classified as “control” were here named cognitively unimpaired (CU), whereas patients with mild cognitive impairment (MCI) and dementia were here grouped into cognitively impaired (CI). Participants were classified for Aβ-positivity based on having an abnormal Aβ PET scan, measured by [^18^F]-florbetapir PET, defined by a global cortical composite with standardized uptake value ratios (SUVr) with average value greater than 1.11—a threshold that has been extensively validated to identify clinical and biologically relevant brain amyloidosis [26, 27].

### Plasma biomarker analysis

For all plasma Aβ42/40, GFAP, and NfL analyses, selected ADNI samples were collected within 3 months of an Aβ PET scan; *n* = 130 (50% Aβ+), cognitively normal *n* = 54 (37% Aβ+), mild cognitive impairment *n* = 54 (46% Aβ+), and AD *n* = 22 (91% Aβ+). ADNI blood samples are collected in 10 mL K2-EDTA tubes and centrifuged within 1 h of collection at room temperature and centrifuged at 1300*g* for 10 min to obtain the plasma fraction. All plasma samples were frozen on dry ice within 90 min of collection at ADNI sites, shipped to the Biomarker Core laboratory, aliquoted into 0.5 mL polypropylene tubes, and stored at − 80 °C (for detailed information see www.adni-info.org and adni.loni.usc.edu).

Plasma p-tau181 was measured on Simoa HD-X instruments (Quanterix, Billerica, MA, USA) in April 2020 at the Clinical Neurochemistry Laboratory, University of Gothenburg, Mölndal, Sweden [15]. To select the biomarker to represent the plasma Aβ values, we initially compared a total of six plasma Aβ42/40 measures: three mass spectrometry methods (Shimadzu, University of Gothenburg, Washington University) and three immunoassay methods [Simoa Neuro 4-plex E (Quanterix), Simoa Aβ40 and Aβ42 Advantage Kit, Elecsys Neuro Toolkit] analyzed between December 2020 to April 2021; samples were tested in a blinded fashion with analytical controls by the different laboratories (for detailed information on sample handling procedures, assay protocols, and performance, see www.adni-info.org and adni.loni.usc.edu). For this, we evaluated the plasma amyloid biomarkers’ performance to predict Aβ PET positivity by comparing single biomarker-based receiver operating characteristics (ROC) curves with DeLong tests. The plasma Aβ42/40 test with the highest area under the curve and the best performing commercially available assay were then selected for fitting logistic regression models in the next analysis stage.

### Statistical analysis

Demographic information was compared between groups with *t tests* for continuous variables and *Χ*^2^ tests for categorical variables. Using a single-biomarker ROC curve approach, we compared the area under the curve (AUC) for each biomarker for cross-sectionally identifying patients with cerebral amyloidosis. To assess the robustness of these biomarkers individually, we repeated these analyses by introducing random variations in the original biomarker measurements, ranging from 1% to 20% in ± 1% intervals.

Next, we evaluated the power of different biomarker combinations to predict Aβ positivity with a logistic regression framework. For this, an initial basic demographic model was built including only age, sex, and *APOE*-ε4 carriership status as predictors of Aβ-positivity status. Then, we evaluated logistic regression models with the addition of the four biomarkers (Aβ42/40 = “A”, GFAP = “G”, p-tau181 = “P” and NfL = “N”) in all possible combinations: basic demographic model plus only one biomarker; basic demographic model plus different combinations of two or three biomarkers (e.g., AP; AGP); basic demographic model plus all four biomarkers (e.g., AGNP). To identify which specific biomarkers were the best predictors of brain amyloidosis, we evaluated models based on a decision tree, schematically represented in Fig. 2. Among all models, the best-fitting model was defined as the one with the lowest Akaike information criterion (AIC) value. Then, we performed likelihood ratio (LR) tests between the best-fitting model and those models with up to two AIC units above that of the best-fitting, leading to exclusion of models significantly inferior to the best-fitting model. Among the remaining models, the most useful biomarker combination was considered as the one present in the model with the lowest Schwartz’s Bayesian information criterion (BIC), a more stringent metric than the AIC [28]. This process was repeated for logistic regression models fitted in three different populations across the AD continuum [all participants (*n* = 118; also had CU/CI status as covariate in the model), CU (*n* = 50), and CI (*n* = 68)]. This was firstly performed for IP-MS Aβ42/40 from Washington University as “A^IP-MS^”, and in an additional analysis, the Simoa Neuro 4-plex E Aβ42/40 was utilized as the alternative “A^Simoa^” in the models.

For assessing the robustness of these model selections, we repeated these analyses by introducing random variations to the original biomarker measurements. Firstly, we tested robustness using the reported coefficients of variance (CVs) for each analytical technique [A^IP-MS^, CV = 4.0%^7^; p-tau181^15^, CV = 6.6%; (Supplementary Table 1, NfL: CV = 1.2%, A^Simoa^, CV = 1.0%; GFAP, CV = 10.5%)], and secondly, we tested robustness using random variations of ± 5%, ± 10%, ± 15%, and ± 20% of the original biomarker values. Each robustness analysis was repeated in 10 iterations. We then applied the above-described decision tree to each iteration to assess whether the analytical variance could result in the selection of different biomarker combinations. Continuous predictors were centered and log-transformed depending on their distribution. All analyses were performed with R Statistical Software (https://www.r-project.org/). Statistical significance was set as *α* = 0.05, and all tests were two-tailed.

## Results

### Study participant characteristics

The demographic characteristics of the study participants are displayed in Table 1. In the full sample (*n* = 118), Aβ-positivity was confirmed by Aβ PET in *n* = 60 (50.8%) of individuals. Aβ-negative and Aβ-positive groups were evenly distributed for age, gender, and years of education. As expected, a significantly increased prevalence of patients with cognitive decline (*P* < 0.05), *APOE*-ε4 carriage status (*P* < 0.01), and poorer MMSE (*P* < 0.0001) was observed in the Aβ-positive group.

**Table 1:** Demographics of selected participants from the ADNI cohort

	Aβ PET negative (*n* = 58)	Aβ PET positive (*n* = 60)	*P* value
Age, years, median (IQR)	70.8 (66.5, 75.7)	73.8 (69.9, 77.4)	0.14
Clinical diagnosis, *n* (CU/CI)	30/28	20/40	0.04
Female, *n* (%)	24 (41.4%)	26 (43.3%)	0.98
Years of education, median (IQR)	18.0 (14.2, 18.0)	16.0 (14.0, 18.0)	0.33
*APOE*-ε4 carriers, *n* (%)	15 (25.9%)	32 (53.3%)	< 0.01
MMSE score, median (IQR)	29.0 (28.0, 30.0)	27.5 (24.0, 29.2)	< 0.0001
Florbetapir, global SUVR, median (IQR)	1.00 (0.954, 1.03)	1.33 (1.22, 1.46)	< 0.0001
Aβ_42/40_ IP-MS, median (IQR)	0.132 (0.128, 0.141)	0.122 (0.117, 0.127)	< 0.0001
Aβ_42/40_ Simoa, median (IQR)	0.050 (0.043, 0.054)	0.044 (0.040, 0.048)	< 0.01
GFAP, pg/mL, median (IQR)	113 (80.7, 154)	164 (125, 223)	< 0.001
P-tau181, pg/mL, median (IQR)	11.7 (8.2, 17.2)	18.8 (13.1, 23.0)	< 0.01
NfL, pg/mL, median (IQR)	23.6 (17.7, 36.1)	31.5 (24.8, 40.1)	0.04

Data shown as median (IQR; interquartile range) or *n* (%), as appropriate. Continuous variables were compared using *t* test and Pearson’s chi-square to compare frequencies of categorical variables between groups. As further explained, Aβ42/40 IP-MS corresponds to the IP-MS assay from Washington University whilst Aβ42/40 Simoa refers to the measurements from the Simoa Neuro 4-plex E assay

*Abbreviations*: *Aβ* amyloid-β, *CU* cognitively unimpaired, *CI* mild cognitive impairment, *MMSE* Mini-Mental State Examination, *NfL* neurofilament light chain, *P-tau181* tau phosphorylated at threonine 181, *SD* standard deviation, *SUVR* standardized uptake value ratio

### Comparison of plasma Aβ42/40 methods to identify Aβ PET burden

Our first task was to select a plasma Aβ42/40 method to represent “A” in our models. Data from six plasma  Aβ42/40 assays were included in the ADNI database (Supplementary Figure 1). We determined that the IP-MS assay from Washington University discriminated Aβ-positive and Aβ-negative groups with the highest AUC and was selected as the “A^IP-MS^” variable in our models (AUC = 83.1%; 95% CI 75.5–90.7%; Supplementary Figure 2A). This method was found to be statistically superior to the other five Aβ42/40 assays included (DeLong test, Shimadzu, *P* = 0.007; University of Gothenburg, *P* = 0.0006; Simoa Neuro 4-plex E, *P* = 0.001; Simoa Aβ40 and Aβ42 Advantage Kit, *P* = 0.0006, Roche Elecsys, *P* = 0.03). In addition, we aimed to have a commercially available immunoassay as an alternative “A” to run the sensitivity analysis. We thus compared Aβ42/40 measured with Simoa Neuro 4-plex E (AUC = 65.1%; 95% CI 55.2–75.1%) with Aβ42/40 measured with Simoa Aβ40 and Aβ42 Advantage Kit (AUC = 55.3%; 95% CI 44.5–65.8%). We found no statistical difference between these AUCs (DeLong test: *P* = 0.41; Supplementary Figure 2B), and therefore, for practical reasons, we chose to perform the sensitivity analysis using Aβ42/Aβ40 measured with Simoa Neuro 4-plex E “A^Simoa^”, as GFAP and NfL are quantified in the same multiplex assay.

### Robustness of individual plasma biomarkers for Aβ-positivity

We evaluated how well the biomarkers identify participants’ Aβ status (for biomarker distribution by Aβ status see Supplementary Figure 3). All plasma biomarkers were significantly altered between Aβ-positive and Aβ-negative groups (Aβ42/40^IP-MS^ < 0.0001; Aβ42/40^Simoa^ < 0.01; GFAP< 0.001; p-tau181 < 0.01; NfL = 0.04). We then investigated how their AUC is affected by adding random variations on its original values. This robustness analysis sought to investigate if biomarkers’ performance would remain constant if the values were to change within a given CV. The rationale is that levels for plasma biomarkers may vary across analytical runs, laboratories, and cohorts [29], but data on this potential issue is essentially lacking for these biomarkers. This analysis was firstly done including all participants but also within CU and CI groups separately.

**Fig. 1:**
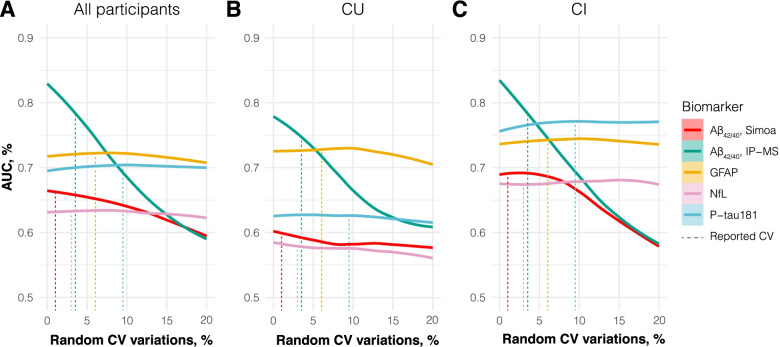
Robustness of the individual biomarkers at distinguishing Aβ status. The line plot shows the AUC for each individual biomarker at each random CV variations, ranging from up to and including 1 to 20% variations of the original biomarker measurements (represented here at 0%). This analysis was performed including all participants (**A**) as well as within CU (**B**) and CI (**C**) groups. Abbreviations: AUC, area under the curve; CI, cognitively impaired; CU, cognitively unimpaired; CV, coefficient of variation

When all participants were evaluated, A^IP-MS^ had the highest AUC (AUC = 83.1%; 95% CI 75.5–90.7%), followed by GFAP (AUC = 71.7%; 95% CI 62.4–81.0%) and p-tau181 (AUC = 69.4%; 95% CI 59.6–79.3%; Fig. 1A). However, with increased CV variation, the “predictive” power of A^IP-MS^ was drastically affected—while GFAP and p-tau181 AUCs remained stable through to a simulated CV of 20%. Results from the CU group followed similar pattern to what was observed for the analysis with all participants (Fig. 1B). Differently, for the CI group, original biomarker values concluded that A^IP-MS^ have the highest AUC followed by p-tau181 and then GFAP (Fig. 1C). However, A^IP-MS^ “predictive” power was strongly impacted with even smaller variations on the CV (< 5%) as compared to what described in the analysis with all participants.

### Identifying Aβ-positivity using biomarker models

**Fig. 2:**
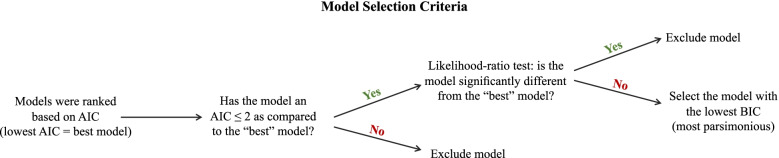
Model selection criteria. The decision tree shows the steps that were followed when deciding the best biomarker model in each of the analyses. AIC, Akaike information criterion; BIC, Bayesian information criterion

The decision tree criteria for selecting biomarker models are illustrated in Fig. 2. This criterion firstly assessed models by AIC value and then LR tests between the best-fitting model and those models within two AIC units. Models significantly different from the best-fitting model were then rejected. Among the remaining models, the most useful biomarker combination was considered as the one present in the model with the lowest BIC.

In all participants, the model “A^IP-MS^G” was selected as the superior model and demonstrated high accuracy for identifying Aβ-positivity (AUC = 86.5; 95% CI 79.7–93.4%; Table 2). Models “A^IP-MS^GP” and “A^IP-MS^GN” were < 2 AIC units of the selected model and were not statistically different to “A^IP-MS^G” in LR tests (*P* > 0.5). However, these three-biomarker models were > 4 BIC units away from “A^IP-MS^G”. The highest-ranking single biomarker model “A^IP-MS^” was shown to be > 2 AIC units from the selected model “A^IP-MS^G”. In CU participants, model “A^IP-MS^” was the model selected by our criteria (AUC = 82.3; 95% CI 68.5–96.1%; Table 2). Models “A^IP-MS^P”, “A^IP-MS^N”, “A^IP-MS^G”, and “A^IP-MS^GN” were < 2 AIC units of the selected model and were not statistically different to “A^IP-MS^” in the LR test (*P* > 0.3). However, these biomarker models were > 2 BIC units away from “A^IP-MS^”. In CI participants, the model “A^IP-MS^GP” was selected by our criteria and demonstrated the highest overall accuracy for identifying Aβ-positivity in our study (AUC = 93.5; 95% CI 87.5–99.5%; Table 2). Only the four-plasma biomarker model “A^IP-MS^GNP” was within 2 AIC units from the best model and was not statistically different to “A^IP-MS^GP” in LR test (*P* = 0.18). However, “A^IP-MS^GNP” was > 4 BIC units away from “A^IP-MS^GP”. In summary, our criteria selected “A^IP-MS^GP”, “A^IP-MS^”, and “A^IP-MS^GP” for identifying Aβ-positivity in all participants, CU participants, and CI patients, respectively.

**Table 2:** Summary information of the selected biomarker models (original measurements)

	Model	AIC	BIC	*R*^2^, unadjusted	*R*^2^, adjusted	AUC, 95% CI	LRT x^2^	*P* value
**IP-MS for Aβ42/40**								
All participants	AG	124.2	143.6	40.7%	37.5%	86.5% (79.7, 93.4)	53.3	< 0.0001*
CU	A	62.6	76.7	33.5%	25.9%	82.3% (68.5, 96.1)	16.7	< 0.001*
CI	AGP	59.2	77.0	58.7%	52.9%	93.5% (87.5, 99.5)	43.5	< 0.0001*
**Simoa for Aβ42/40**								
All participants	G	145.5	159.4	22.2%	19.4%	77.5% (68.9, 86.1)	28.0	< 0.0001*
CU	G	71.9	81.4	11.0%	3.1%	72.8% (58.1, 87.6)	5.4	0.04
CI	GP	71.7	85.0	42.2%	37.6%	87.1% (78.4, 95.9)	32.4	< 0.01*
**Demographic**								
All participants	–	155.6	166.7	12.7%	10.5%	70.7% (61.3, 80.0)	–	–
CU	–	74.0	81.7	2.4%	− 4.0%	58.1% (41.9, 74.3)	–	–
CI	–	78.9	87.8	27.8%	24.5%	81.3% (71.0, 91.5)	–	–

*Abbreviations*: *AIC* Akaike information criterion, *AUC* area under the curve, *BIC* Bayesian information criterion, *CU* cognitively unimpaired, *CI* mild cognitive impairment, *LRT* likelihood ratio test

*P value of the likelihood ratio test comparing the selected model with the demographic model on the respective sample group

We also performed the sensitivity analysis by replacing “A^IP-MS^” by a commercially available immunoassay for Aβ42/40 “A^Simoa^”, as previously described, which greatly impacted our results. In all participants, it was shown that “G” was the best model based on our criteria (Table 2). The model “G” had a modest accuracy for Aβ-positivity (AUC = 77.5; 95% CI 68.9–86.0). The AIC criteria ranked the model “GP” as the best fitted model and models defined as “G”, “A^Simoa^G”, “A^Simoa^GP”, and “GNP” were within 2 AIC units and not statistically different to “GP” in the LR tests (*P* > 0.05). However, the BIC favored the single biomarker model “G” (159.4) rather than “GP” (161.2) to be best fitting model. In CU participants, the model “G” was also selected as the superior model for Aβ-positivity (AUC = 72.8; 95% CI 58.1–87.6). Only “GN” was within 2 AIC units and was not statistically different to “G” (*P* = 0.29) but > 2 BIC units from the best fitted model. In CI participants, the model “GP” was selected as the superior model Aβ-positivity (AUC = 87.1; CI 78.4–95.9). Models “A^Simoa^GP” and “GNP” were within 2 AIC units and were not statistically different to “GP” (*P* > 0.25). In summary, when using immunoassay instead of IP-MS determinations for “A”, our criteria selected “G” for predicting Aβ-positivity in all participants and CU participants. The model “GP” was selected for predicting Aβ-positivity in CI participants.

### Comparing selected models for Aβ-positivity

We compared the selected models from each category (all participants, CU participants and CI participants) from the analysis which included “A^IP-MS^” versus the analysis which used immunoassays for “A^Simoa^”. In two scenarios (all participants and CI participants), models that included “A^IP-MS^” statistically outperformed the equivalent analysis without; all participants (“A^IP-MS^G” versus “G”, *P* = 0.017) and CI participants (“A^IP-MS^GP” versus “GP”, *P* = 0.042). In CU participants, no statistical superiority was observed (“A^IP-MS^” versus “G”, *P* = 0.20).

### Robustness of plasma biomarkers models for Aβ-positivity

Next, after demonstrating that certain biomarkers models have superiority in determining Aβ status at different stages of the disease, we sought to perform a robustness analysis to investigate if the selected models would remain constant if the biomarker values were to change within a given simulated CV. We performed 10 iterations of randomly changed values for each one of the assays (Aβ42/40 defined as a single assay). Firstly, we changed biomarker values within and up to the reported CV of each assay (see methods), and secondly, we changed biomarker values within and up to 5%, 10%, 15%, and 20%—anticipating larger variations in multi-laboratory comparisons. The same model selection decision tree (Fig. 2) was then applied to each robustness iteration.

**Fig. 3:**
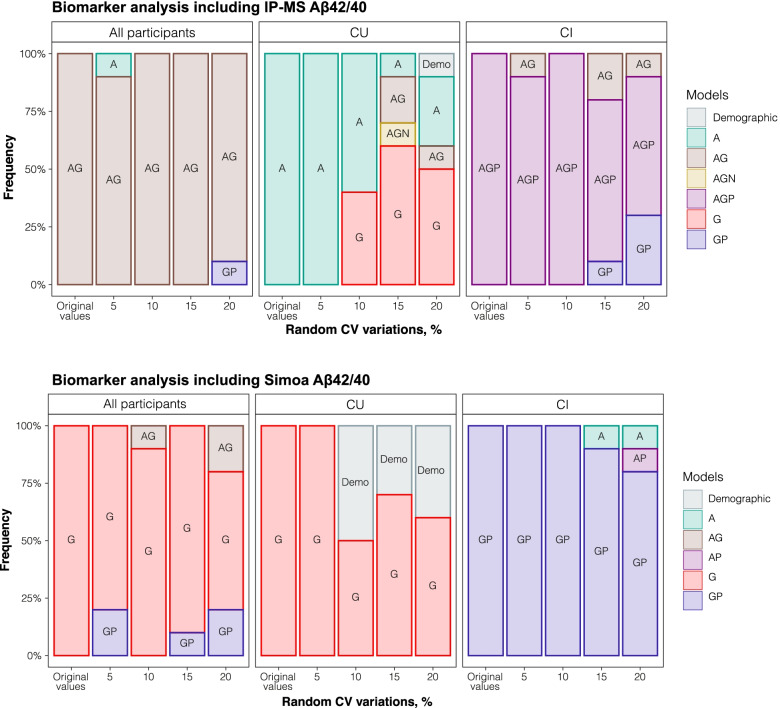
Model selection over random variation. Stacked bar chart shows the frequency that each model was selected when random CV variation was generated for each biomarker. The best model selected when using the original biomarker measurements is represented on the “Original values” bar. The following bars represent the frequency that a model was selected as “best model,” at each of the 10 iterations, when random CV variation was created ranging from 5 to 20%. The analysis was performed with all participants, within CU and within CI groups. A, plasma Aβ42/40; AG, plasma Aβ42/40 + GFAP; AGN, plasma Aβ42/40 + GFAP + NfL; AGP, plasma Aβ42/40 + GFAP + p-tau181; AUC, area under the curve; CI, cognitively impaired; CU, cognitively unimpaired; CV, coefficient of variation; G, plasma GFAP; GP, plasma GFAP + p-tau181

Overall, the robustness of the reported variations did not largely impact on the model selection (Supplementary Table 1). However, if an increased variation up to 10% (or greater) was applied, the model selection shifted from “A^IP-MS^” to “G” in CU participants (Fig. 3A). Limited change in biomarker selection was seen for all and CI participants, with some deviation when CV varied at 15–20%.

## Discussion

In this study, our results denote that plasma Aβ42/40 as determined by IP-MS was the best predictor of Aβ-positivity, followed by p-tau181 and GFAP. In a novel approach, preparing for such tests in clinical chemistry routine, we were interested in how variations in the biomarker measurements would impact the robustness of these biomarker performances. Random variations on the CV indicated that, around a simulated CV of 5%, the accuracy of IP-MS Aβ42/40 drops below to that of GFAP and p-tau181. In contrast, GFAP and p-tau181 performances remain stable even at a 20% CV. When biomarkers were evaluated in several combinations of models, IP-MS Aβ42/40 was the most significant contributor in predicting Aβ-positivity at the preclinical stages of AD, and adding p-tau181, GFAP, or NfL did not significantly improve this finding. At the CI stages of the AD continuum, however, a model combing IP-MS Aβ42/40, GFAP, and p-tau181 was found to be the best indicator of Aβ-positivity and results in very high accuracy. In general, models that included IP-MS Aβ42/40 significantly outperformed model selections that included Simoa Aβ42/40 as an alternative. We then investigated how the variations in biomarker CV would impact on the optimal model selection. With small variations in biomarker measurements, all selected models were preserved and shown to be robust. However, for CU participants, IP-MS measurements were not able to withstand a larger variation (CV > 10%), being subsequently replaced by GFAP in the majority of model iterations. Originally selected Aβ-positivity models which included all participants and CI were robust, i.e., were most frequently selected, up to 15%.

The use of plasma biomarkers to highlight underlying cerebral Aβ pathology is greatly anticipated in clinical routine and disease-modifying trials, for both symptomatic and preclinical stages of AD. An increasing number of plasma biomarkers, shown to be related to Aβ pathology, have now been reported [7, 15, 30, 31], but it is yet to be determined which combinations are best suited in a heterogeneous population (e.g., diagnosis independent), preclinical or symptomatic stages. In this study, we show that IP-MS Aβ42/40 have high accuracy in the detection of Aβ pathology at all stages of the AD continuum and, in combination with GFAP and p-tau181, had a very high accuracy to determine Aβ-positivity in CI (> 93%). There is a mixture of reports about the use plasma Aβ42/40 in the literature [32]. While immunoassay results of p-tau from differing platforms are seemingly concordant with reproducible results and measures of plasma NfL and GFAP tend to utilize the same Simoa technology [33], methods to determine plasma Aβ42/40 varies. This study shows the importance of method choice for the detection of brain amyloidosis by plasma Aβ since, when IP-MS measures of Aβ42/40 were not included, Aβ-positivity was best represented by GFAP and p-tau181 and not by immunoassay determinations of Aβ42/40. It is also important to signify that models that included IP-MS significantly outperformed models without it.

It is unlikely that Aβ PET will be replaced from the recruitment process in anti-Aβ trials, as target engagement and possible termination of Aβ removal agents are necessary to determine participant’s baseline and subsequent changes in Aβ burden relative to the intervention process [4]. However, the plasma biomarker models demonstrated in this study may act, with good accuracy, as important initial screening tools to enrich a population for a larger success rate of Aβ PET scan or tau PET scans [4] in the recruitment process. Our aim was to report the best plasma models for this process while acknowledging that IP-MS technology currently has constraints on availability and costs in comparison to semi-automated immunoassay methods. Thus, we included a commercially available immunoassay which did not significantly add to any biomarker model and was inferior to IP-MS Aβ42/40, GFAP, and p-tau181 at the single biomarker level. Therefore, at this time, it is important to disseminate that IP-MS Aβ42/40 measurements cannot simply be replaced by immunoassay Aβ42/40 and, if IP-MS is not a viable option, Aβ-positivity is best represented by surrogate measures of Aβ pathology, e.g., GFAP and p-tau181, as shown in this study. This difference between Aβ methods could be explained by IP-MS being less prone to matrix effects that are particularly noticeable in complex biological fluids such as blood.

However, there are constraints to Aβ42/40 as a plasma biomarker which could be significant limiting factor in clinical chemistry routine. As Aβ42/40 is suggested to change by only 10% in Aβ-positivity individuals, compared with 50% in CSF [7], a moderate change in assay variability could greatly influence the result. Our first robustness analysis, which focused on random variation (not bias) on the single biomarker level, denoted a diminishing performance of IP-MS Aβ42/40 as the CV increased. While IP-MS Aβ42/40 was the best performing biomarker, random variations ~ 5% lowered the accuracy below GFAP in CU participants and p-tau181 in CI participants. As the CV increased to 15%, an accepted level of intra-assay variation in clinical chemistry, IP-MS Aβ42/40 produced AUC’s only around 60% to predict Aβ-positivity. In contrast, GFAP and p-tau181 maintained the same level of accuracy regardless of intra-assay variation. This demonstrates that plasma measures Aβ42/40 need to have a very low-level variability in order to maintain maximum accuracy. Given the more complex nature of IP-MS protocol and heterogeneous sample collections, we feel that an analytical variability of 10% or higher is likely across laboratories, particularly in ad hoc sampling in routine testing. While simulated variations showed clear shifts of best performance for single biomarkers, models incorporating biomarker combinations were more robust, remaining relatively stable with greater variations—IP-MS Aβ42/40 in combination with either GFAP (all participants) and p-tau and GFAP (CI) were relatively robust up to 20%. Again, however, in CU, where IP-MS Aβ42/40 alone was the best biomarker, higher variability affects this model selection, opting for GFAP at > 10% CV.

Despite both being antibody-based assays, the Simoa and IP-MS Aβ assays have somewhat different biochemical properties. However, it is unknown if these technical differences contribute to the observed performances. The Simoa assay utilizes the same principle as a sandwich immunoassay, where the target analyte is first bound by a capture antibody and this immunocomplex further refined by binding of a detection antibody following washing steps to remove unspecific binding. In the Simoa Aβ40 and Aβ42 assays, the same capture antibody common to both analytes is used while antibodies specific to either peptide are used for detection [34]. The Aβ40 and Aβ42 assays in the Simoa Neuro 4-plex E kit and Advantage kit are based on the same biochemical principle except that (1) different Aβ antibodies are used in either kit, and (2) the latter kit provides multiplexing advantages that allow Aβ40 and Aβ42 to be measured alongside NfL and GFAP concurrently in the same sample. The IP-MS assay enriches for Aβ in plasma by precipitating the analyte signal by binding to an Aβ-specific antibody or a cocktail of Aβ antibodies coated onto paramagnetic beads. Following elution of the bound analytes, the signal is read with a mass spectrometer, using labeled synthetic peptides as quantification [32] standards. The biochemistry of different plasma Aβ assays have been summarized in a recent review [32].

## Limitations

The foremost constraint in this study is that the sample size of the ADNI participants with all plasma biomarkers was limited (total, *n* = 118; CU, *n* = 50; CI, *n* = 68), which could have led to slightly reduced overall biomarker performance. Furthermore, it is known that preanalytical procedures and protocol variations may affect biomarker analysis and results, and therefore, we strongly advise the replication of these findings in larger independent cohorts with these available biomarker methods. However, we are encouraged that these results are in line with developing evidence from the recent literature [30]—namely, IP-MS Aβ being a strong predictor of amyloidosis [6, 7], particularly at CU [6] and p-tau181 being more important at CI [15]. In studies where IP-MS Aβ has not been included, GFAP has emerged as the principal candidate for amyloidosis [18, 19, 31, 35]. It must be noted that plasma p-tau217 and p-tau231 are variables not included in the ADNI cohort at this time. These additional p-tau biomarkers have both been shown to have high accuracy, together with a high fold change in AD, in determining Aβ pathology at both the preclinical and symptomatic phases of the disease and therefore may significantly contribute to the model selections, if available [10, 36].

## Conclusion

In this report, utilizing participants in the ADNI database, we demonstrate that plasma Aβ, as indexed by IP-MS, is the simplest model that best determines Aβ burden at the preclinical stage. At the symptomatic phase, IP-MS Aβ in combination with GFAP and p-tau181 was found to be the simplest model with the highest accuracy. However, the accuracy of plasma IP-MS Aβ42/40 to indicate Aβ burden deteriorates with only a modest increase in analytical variation, which will pose as an issue in ad hoc testing in clinical routine or multicenter laboratory testing in trials. In contrast, despite lower overall accuracies, GFAP and p-tau181 are highly robust. In the absence of IP-MS Aβ measures, GFAP is the best predictor of amyloidosis at the preclinical stage of AD and, in combination with p-tau181, best predicts amyloidosis at the symptomatic phase of the disease.

## Supplementary Information

**Additional file 1 **: **Supplementary Table 1**. Best models selected in the robustness analyses (within reported CV). **Supplementary Figure 1**. Plasma Aß42/40 distribution by groups. **Supplementary Figure 2**. Plasma Aß42/40 discriminative power. **Supplementary Figure 3**. Plasma biomarkers distribution by Aß status.

## Data Availability

The original data used in this manuscript is available for download in the ADNI database (http://adni.loni.usc.edu).
