# Supplementary material for: The accuracy and robustness of plasma biomarker models for amyloid PET positivity
Source: Alzheimers Res Ther. 2022 Feb 7;14:26. doi: 10.1186/s13195-021-00942-0 (PMC8819863; doi:10.1186/s13195-021-00942-0)
Supplement: Supplementary file 1 — Additional file 1 : Supplementary Table 1. Best models selected in the robustness analyses (within reported CV). Supplementary Figure 1. Plasma Aß42/40 distribution by groups. Supplementary Figure 2. Plasma Aß42/40 discriminative power. Supplementary Figure 3. Plasma biomarkers distribution by Aß status. [file 13195_2021_942_MOESM1_ESM.docx]

**Supplementary Material**

**The accuracy and robustness of plasma biomarker models for amyloid PET positivity**

Andréa L. Benedet^1,2*^, Wagner S. Brum^1,3*^, Alzheimer’s Disease Neuroimaging Initiative^🟊^, Thomas K. Karikari^1,4^, Eduardo R. Zimmer^3,5,6^, Henrik Zetterberg^1, 7, 8, 9, 10^, Kaj Blennow^1,7^, Nicholas J. Ashton^1,11,12,13^

**Affiliations**

^1^ Department of Psychiatry and Neurochemistry, Institute of Neuroscience and Physiology, The Sahlgrenska Academy, University of Gothenburg, Sweden; ^2^ Translational Neuroimaging Laboratory, McGill Centre for Studies in Aging, McGill University, Montreal, Quebec, Canada; ^3^ Graduate Program in Biological Sciences: Biochemistry, Universidade Federal do Rio Grande do Sul (UFRGS), Porto Alegre, Brazil; ^4^ Department of Psychiatry, University of Pittsburgh, PA, USA; ^5^ Department of Pharmacology, UFRGS, Porto Alegre, Brazil; ^6^ Graduate Program in Biological Sciences: Pharmacology and Therapeutics, UFRGS, Porto Alegre, Brazil; ^7^ Clinical Neurochemistry Laboratory, Sahlgrenska University Hospital, Mölndal, Sweden; ^8^ Department of Neurodegenerative Disease, UCL Institute of Neurology, London, UK; ^9^ UK Dementia Research Institute at UCL, London, UK; ^10^ Hong Kong Center for Neurodegenerative Diseases, Hong Kong, China; ^11^ Wallenberg Centre for Molecular and Translational Medicine, Department of Psychiatry and Neurochemistry, Institute of Neuroscience and Physiology, the Sahlgrenska Academy at the University of Gothenburg, Sweden; ^12^ King’s College London, Institute of Psychiatry, Psychology & Neuroscience, Maurice Wohl Clinical Neuroscience Institute, London, UK; ^13^ NIHR Biomedical Research Centre for Mental Health & Biomedical Research Unit for Dementia at South London & Maudsley NHS Foundation, London, UK.

^*^These authors contributed equally to this work.

^🟊^Data used in the preparation of this article were obtained from the Alzheimer’s Disease Neuroimaging Initiative (ADNI) database (http://adni.loni.usc.edu/). As such, the investigators within the ADNI contributed to the design and implementation of ADNI and/or provided data but did not participate in analysis or writing of this report. A complete listing of ADNI investigators can be found at

http://adni.loni.usc.edu/wp-ontent/uploads/how_to_apply/ADNI_Acknowledgement_List.pdf.

Corresponding author:

**Dr. Andréa L. Benedet**

Clinical Neurochemistry Laboratory, Sahlgrenska University Hospital, Mölndal, Sweden, and the Department of Psychiatry and Neurochemistry, Institute of Neuroscience and Physiology, The Sahlgrenska Academy, University of Gothenburg, SE 43180, Gothenburg, Sweden

Email: [andrea.benedet@gu.se](mailto:andrea.benedet@gu.se)

**Supplementary Tables**

**Supplementary Table 1.** **Best models selected in the robustness analyses (within reported CV)**

| **Sample** | Models within two AIC units of the lowest AIC value | | |
| --- | --- | --- | --- |
|  | **Whole sample** | **CU** | **CI** |
| **IP-MS for Aβ42/40** |  |  |  |
| Original sample | **AG**, AGP, AGN | **A**, AG, AGN, AP, AGP, AN | **AGP**, AGNP |
| Robustness iteration 1 | **AG**, AGP, AGN | **A**, AP, AN, AG | **A**, AGP, AGNP |
| Robustness iteration 2 | **AG**, AGN, AGP | **A**, AP, ANP, AN, AGNP, AGN, AGP | **AGP**, AGNP |
| Robustness iteration 3 | **AG**, AGP, AGN | **A**, AP, AG, AN | **AGP**, AGNP |
| Robustness iteration 4 | **AG**, AGP, AGN | **A**, AN, AP, AG, AGN | **AGP**, AGNP |
| Robustness iteration 5 | **A**, AG, AGN, AGP | **A**, AN, AGN, ANP, AP | **AGP**, AGNP |
| Robustness iteration 6 | **AG**, AGP, AGN | **A**, AN, AGN, AP, AG | **AGP**, AGNP |
| Robustness iteration 7 | **AG**, AGN, AGP | **A**, AN, AP, AG, AGN | **AGP**, AGNP |
| Robustness iteration 8 | **AG**, AGP, AGN | **A**, AP, AG, AN | **AGP**, AGNP |
| Robustness iteration 9 | **AG**, AGP, AGN | **A**, AP, AG, AN, AGN | **AGP**, AGNP |
| Robustness iteration 10 | **AG**, AGP, AGN | **A**, AN, AP, AG, AGN | **AGP**, AGNP |
|  |  |  |  |
| **Simoa for Aβ42/40** |  |  |  |
| Original sample | **G**, GP, AGP, AG, GNP | **G**, GN, GP | **GP**, AGP, GNP |
| Robustness iteration 1 | **G**, AGP, GP, AG, GNP, AGNP | **Demographic**, G, GN, A, GP | **GP**, AGP, ANP, GNP |
| Robustness iteration 2 | **G**, AGP, GP, AG, GNP, AGNP | **Demographic**, G, GN, A, GP | **GP**, AGP, GNP |
| Robustness iteration 3 | **G**, GP, AGP, AG, GNP | **G**, GN, GP | **GP**, AGP, GNP |
| Robustness iteration 4 | **G**, GP, AGP, AG, GNP | **G**, GN, GP | **GP**, AGP, GNP |
| Robustness iteration 5 | **G**, GP, AGP, AG, GNP | **Demographic**, G, GN, A, GP | **GP**, AGP, GNP |
| Robustness iteration 6 | **G**, GP, AGP, AG, GNP | **G**, GN, GP | **GP**, AGP, GNP |
| Robustness iteration 7 | **G**, GP, AGP, AG, GNP | **Demographic**, G, GN, A, GP | **GP**, AGP, GNP |
| Robustness iteration 8 | **G**, GP, AGP, AG, GNP | **Demographic**, G, GN, A, GP | **GP**, AGP, GNP |
| Robustness iteration 9 | **G**, GP, AGP, AG, GNP | **G**, GN, GP | **GP**, AGP, GNP |
| Robustness iteration 10 | **G**, GP, AGP, AG, GNP | **Demographic**, G, GN, A, GP | **GP**, AGP, GNP |

Results of the model selection of each iteration (total 10 iterations) where the biomarker original data were randomly changed within the reported coefficient of variation (CV) of each assay.

Abbreviations: A, plasma Aβ42/40; AIC, Akaike Information Criterion; CI, cognitively impaired; CU, cognitively unimpaired; G, plasma GFAP; N, plasma NfL; P, plasma p-tau181.

**Supplementary Figures**


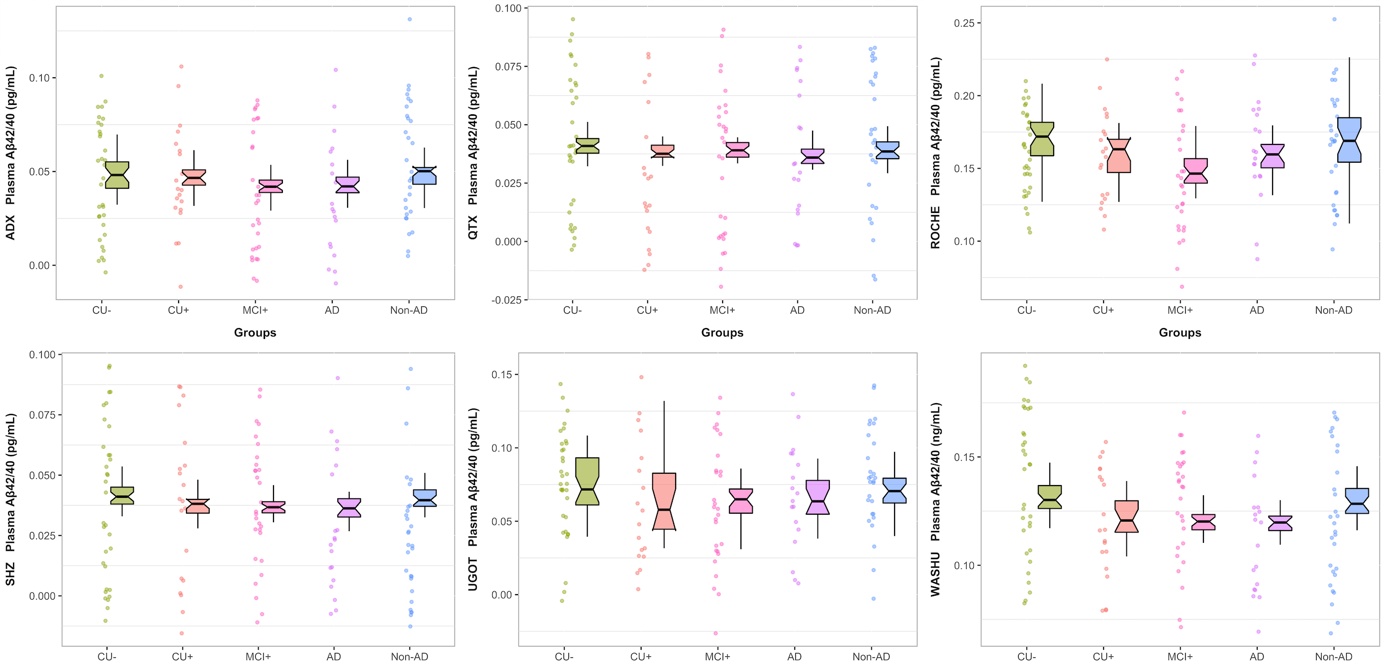


**Supplementary Figure 1. Plasma Aß42/40 distribution by groups.**

Box-plots showing plasma Aß42/40 distribution across groups on the different methodologies tested. The box-plots depict the median (horizontal bar), interquartile range (IQR, hinges) and 1.5 x IQR (whiskers). Amyloid status was given based on ADNI [18F]florbetapir PET published cutoff of 1.11 SUVR and clinical groups followed ADNI diagnostic criteria.

Abbreviations: Aβ, amyloid-β; AD, Alzheimer’s disease (Aβ-positive dementia patients); ADX, ADx Neurosciences Simoa Neuro 4-plex E (Quanterix); CU-, Aβ-negative cognitively unimpaired; CU+, Aβ-positive cognitively unimpaired; MS, Mass Spectrometry assay; MCI+, Aβ-positive mild cognitive impairment; Non-AD, Aβ-negative MCI or dementia patients; ROCHE, Roche Elecsys Neuro Toolkit; SHZ, Shimadzu MS assay; QTX, Quanterix Simoa Aβ40 and Aβ42 Advantage Kit; UGOT; University of Gothenburg MS assay; WASHU; Washington University MS assay.


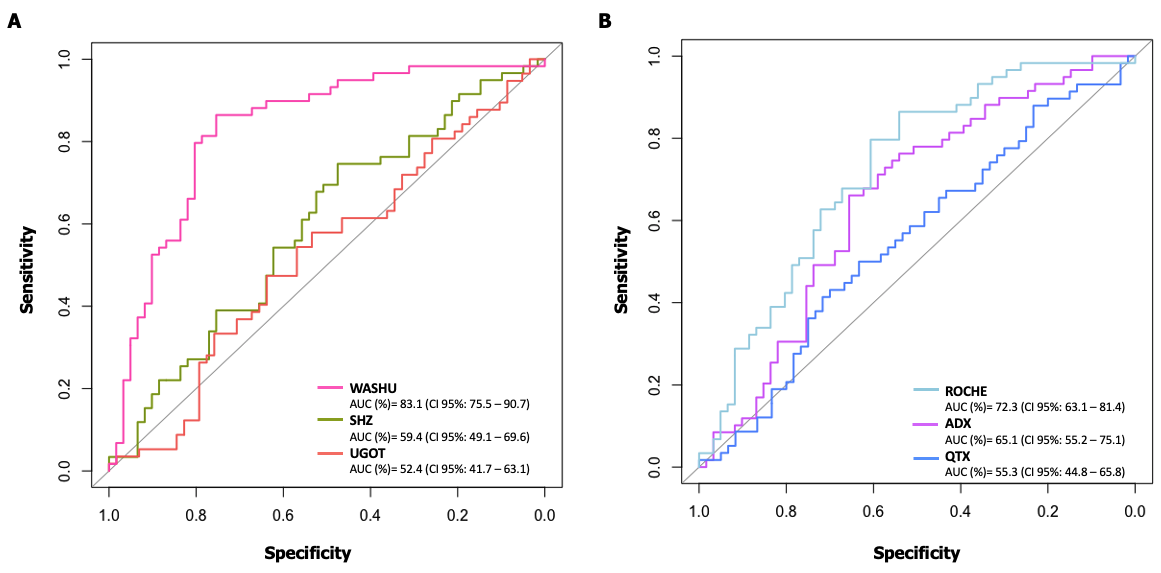


**Supplementary Figure 2. Plasma Aß42/40 discriminative power.**

ROC analysis was performed to test the accuracy of plasma Aß42/40 to discriminate between Aβ-positive and negative individuals using MS based methods (A) and using commercially available immunoassays (B). Amyloid status was given based on ADNI [18F]florbetapir PET published cutoff of 1.11 SUVR.

Abbreviations: Aβ, amyloid-β; ADX, ADx Neurosciences Simoa Neuro 4-plex E (Quanterix); AUC, area under the curve; CI, confidence interval; MS, Mass Spectrometry assay; ROCHE, Roche Elecsys Neuro Toolkit; SHZ, Shimadzu MS assay; QTX, Quanterix Simoa Aβ40 and Aβ42 Advantage Kit; UGOT; University of Gothenburg MS assay; WASHU; Washington University MS assay.


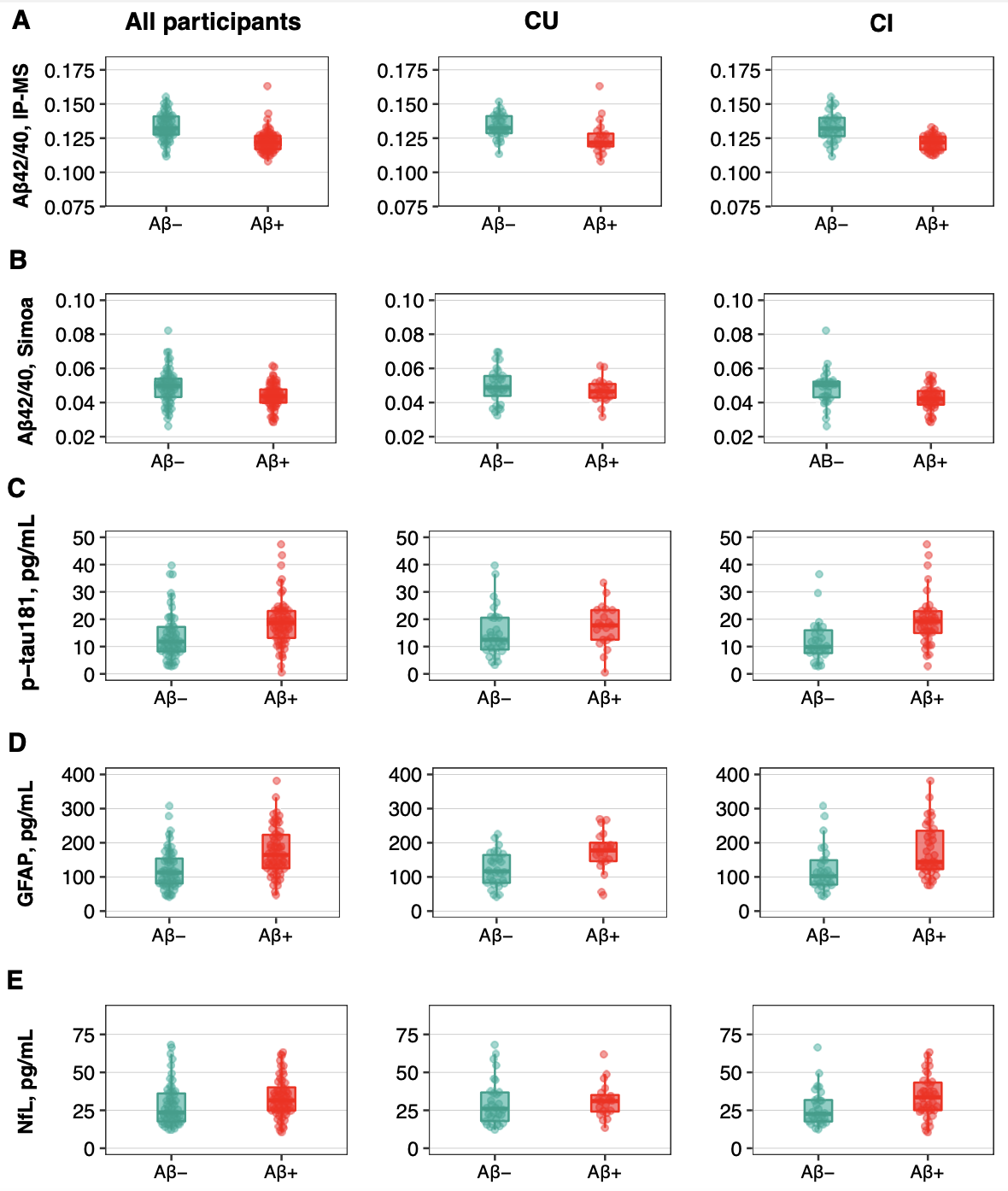


**Supplementary Figure 3. Plasma biomarkers distribution by Aß status.**

Box-plots showing the distribution of plasma biomarkers by Aβ status in all participants as well as in CU and CI groups. The box-plots depict the median (horizontal bar), interquartile range (IQR, hinges) and 1.5 x IQR (whiskers).

Abbreviations: Aβ, amyloid-β.
